# Antigenic and immunosuppressive properties of a trimeric recombinant transmembrane envelope protein gp41 of HIV-1

**DOI:** 10.1371/journal.pone.0173454

**Published:** 2017-03-10

**Authors:** Michael Mühle, Melissa Lehmann, Kerstin Hoffmann, Daniel Stern, Tobias Kroniger, Werner Luttmann, Joachim Denner

**Affiliations:** 1 Robert Koch Institute, Berlin, Germany; 2 ImmunoTools GmbH, Friesoythe, Germany; 3 Division for Regenerative Immunology and Aging, Berlin Center of Regenerative Therapies, Berlin, Germany; 4 Robert Koch Fellow, Robert Koch Institute, Berlin, Germany; University of Liverpool Institute of Infection and Global Health, UNITED KINGDOM

## Abstract

The transmembrane envelope (TM) protein gp41 of the human immunodeficiency virus—1 (HIV-1) plays an important role during virus infection inducing the fusion of the viral and cellular membranes. In addition, there are indications that the TM protein plays a role in the immunopathogenesis leading to the acquired immunodeficiency syndrome (AIDS). Inactivated virus particles and recombinant gp41 have been reported to inhibit lymphocyte proliferation, as well as to alter cytokine release and gene expression. The same was shown for a peptide corresponding to a highly conserved domain of all retroviral TM proteins, the immunosuppressive domain. Due to its propensity to aggregate and to be expressed at low levels, studies comprising authentic gp41 produced in eukaryotic cells are extremely rare. Here we describe the production of a secreted, soluble recombinant gp41 in 293 cells. The antigen was purified to homogeneity and characterised thoroughly by various biochemical and immunological methods. It was shown that the protein was glycosylated and assembled into trimers. Binding studies by ELISA and surface plasmon resonance using conformation-specific monoclonal antibodies implied a six-helix bundle conformation. The low binding of broadly neutralising antibodies (bnAb) directed against the membrane proximal external region (MPER) suggested that this gp41 is probably not suited as vaccine to induce such bnAb. Purified gp41 bound to monocytes and to a lesser extent to lymphocytes and triggered the production of specific cytokines when added to normal peripheral blood mononuclear cells. In addition, gp41 expressed on target cells inhibited the antigen-specific response of murine CD8^+^ T cells by drastically impairing their IFNγ production. To our knowledge, this is the first comprehensive analysis of a gp41 produced in eukaryotic cells including its immunosuppressive properties. Our data provide another line of evidence that gp41 might be directly involved in HIV-1 immunopathogenesis through modulation of the cytokine release and active inhibition of immune responses.

## Introduction

The transmembrane envelope (TM) proteins of retroviruses play an important role during infection of target cells. After interaction of the surface envelope protein gp120 of HIV-1 with its receptors CD4 and CCR5/CXCR4 and intrusion of the fusion peptide of gp41 into the cellular membrane, two helical domains in the gp41 molecule, the N- and C-terminal helical region (CHR and NHR) interact. This brings the cellular and viral membranes in close proximity, and allows fusion pore formation and virus entry (for review see [1, 2]).

Therefore, it is not surprising that gp41 is the target of neutralising and broadly neutralising antibodies (bnAb) preventing infection. BnAb such as 2F5 or 4E10 have been isolated from HIV-1-infected individuals, they are directed against the membrane proximal external region (MPER) of gp41, and neutralise up to 95% of HIV-1 clades. However, despite an enormous effort, until now induction of such bnAb by immunisation with a gp41-derived antigen failed (for review see [3, 4]). To note, peptides corresponding to the helical regions of gp41 were found to prevent fusion and infection by intercalation and such peptides have been used in the clinic for treatment [5].

Since the structural changes of gp41 during infection are extremely complex and difficult to visualize at the molecular level, important information about the fusion process comes from experiments that used monoclonal antibodies directed against various conformational states. For example, the antibody 50–69 recognises an epitope requiring the disulphide bridge between the two cysteines in position 598 and 604 [6, 7]. The antibody D5 specifically binds the fusion intermediate form of gp41 [8] and the antibodies NC-1 and 98–6 recognise the post-fusional six helix bundle (6HB) [6, 9, 10]. Interestingly, a conformation-sensitive binding of monoclonal antibodies recognising linear epitopes in the MPER of gp41 was described [11]. In immunoprecipitation experiments using cells expressing gp120 and gp41 or virus lysates, the bnAb 2F5 showed strong reactivity to prefusogenic gp41, which was largely diminished after triggering the 6HB formation by incubation with soluble CD4. Interestingly, the opposite was true for the monoclonal antibody D50, suggesting that the MPER is prone to structural changes during the fusion process [11].

In the last years evidence has been accumulated showing that gp41 may be involved in HIV-1 induced immunopathogenesis leading to AIDS. In the transmembrane envelope proteins of all retroviruses, including gp41 of HIV-1, a highly conserved domain, the immunosuppressive (Isu) domain, has been identified and it was shown that synthetic peptides corresponding to this domain inhibited mitogen-triggered lymphocyte proliferation, modulated cytokine release and gene expression in human peripheral blood mononuclear cells (PBMCs) (for a review see [12] and references therein). In addition to the studies *in vitro*, important insights into the possible function of retroviral Isu domains were obtained in *in vivo* studies with mice. When immunocompetent mice were inoculated with certain murine tumour cells, these cells were unable to form tumours. Expression of the TM proteins from different retroviruses however allowed the murine cells to produce tumours by efficiently inhibiting the immune system [13–16]. Deletion or point mutations of the Isu domain abrogated the ability of the envelope protein to commit tumour induction [16]. Furthermore, it has been shown that inactivated HIV-1, recombinant gp41 and its corresponding Isu peptide inhibited mitogen-triggered proliferation of human PBMCs, and modulated their cytokine release and gene expression including that of IL-10, IL-8 and IL-6 [17]. Single mutations in the Isu domain of the recombinant TM protein of HIV-1 and of gp41 in the replication competent virus abrogated the induction of IL-10 and IL-6 release [18].

The mechanistic basis for the effects observed for gp41 or the Isu domain is still unknown, and it is also not clear, whether a specific receptor is involved. Using recombinant gp41 produced in bacteria, different binding proteins on the surface of immune human cells were found [19, 20]. Recently, using monomers or polymers of synthetic peptides corresponding to the Isu domain of HIV-1, binding proteins on human lymphocytes were allocated to individual PBMC sub-populations, mainly to monocytes and B-cells [21, 22].

To study its antigenic and immunosuppressive properties in more detail, a recombinant gp41 expressed in human cells was produced and characterised. Its behaviour during gel electrophoresis and binding studies with monoclonal antibodies able to recognise conformational epitopes indicated that the recombinant gp41 is glycosylated, trimeric and in a post-fusional conformation. Furthermore, purified gp41 was effective in modulating cytokine expression on human and even murine immune cells. In addition, gp41 expressed on target cells inhibited the antigen-specific response of murine CD8^+^ T cells.

## Material and methods

### Antibodies

Antibodies recognising conformation-sensitive epitopes in different domains of gp41 were used (see Table 1). Monoclonal antibodies 50–69, F240, D5, 246-D, NC-1, D50, 98–6, 2F5, 4E10, 7H6, 10E8 and Z13E1 were obtained through the NIH AIDS Reagent Program, 5F3 was a kind gift of Hermann Katinger (Polymun Scientific, Austria).

**Table 1:** Kinetic binding rates of monoclonal antibodies to gp41 as measured by SPR.

Antibody	Binding features	*k*_a_ (M^-1^s^-1^)	*k*_d_ (s^-1^)	*K*_D_ (M)
5F3	Oligomer-specific	1.1 × 10^5^	3.0 × 10^−4^	2.8 × 10^−9^
D50	6HB, weak to Pre/Int	8.2 × 10^4^	4.1 × 10^−4^	5.1 × 10^−9^
F240	Linear	7.0 × 10^4^	4.1 × 10^−4^	5.9 × 10^−9^
50–69	Int, 6HB, S-S	5.8 × 10^4^	4.0 × 10^−4^	7.0 × 10^−9^
246-D	Linear	4.7 × 10^4^	4.6 × 10^−4^	9.8 × 10^−9^
98–6	6HB	4.7 × 10^4^	8.1 × 10^−4^	1.3 × 10^−8^
2F5	Pre/Int, weak 6HB	4.0 × 10^4^	7.2 × 10^−4^	1.8 × 10^−8^
NC-1	6HB	4.9 × 10^4^	3.7 × 10^−4^	7.5 × 10^−8^
D5	Int	2.6 × 10^5^	2.8 × 10^−4^	1.1 × 10^−7^
4E10		no binding observed		
7H6		no binding observed		
10E8		no binding observed		
Z13e1		no binding observed		

Abbreviation: 6HB—six-helix bundle, Pre- pre-fusion state, Int—Intermediate state

### Construction of the gp41 expression vector

The sequence coding for the HIV-1 gp41 ectodomain (amino acids 533–684, corresponding to HIV-1 reference strain HXBII, Uniprot entry P04578) was amplified by proof-reading PCR from the HIV infectious molecular clone pNL4-3 using sense primer 5’-GCATGCGGCCGCT**ATCGAGGGAAGG**GCGTCAATGACGCT-3’ and antisense primer 5’-GATCTCTAGACTCGAGTTATAATTTTATATACCACAGCC-3’. The primers introduced an additional factor Xa protease recognition sequence (bold) as well as restriction sites (underlined). The amplicon was introduced into a modified pcDNA3.1 Zeo vector containing an N-terminal CD14 signal peptide and an 8xHis tag sequence using restriction enzymes *Not*I and *Xba*I (Thermo Scientific, USA). To obtain the final expression vector, the complete expression cassette was subcloned into the CMV-driven pIRES neo3 vector (Clontech, USA) by PCR amplification using primers 5'-GCATGAATTCATGGAG-CGCGCGTCC-3' and 5'-GATCGGATCCTTATAATTTTATATACCACAGCCAATTTG-3' (*Eco*RI and *Bam*HI restriction sites underlined). Sequence integrity was verified by sequencing and large-scale plasmid preparations for subsequent transfections were performed using the NucleoBond Xtra Midi kit (Macherey-Nagel, Germany) according to the manufacturer's instructions.

### Generation of 293 cells stably expressing gp41

293 cells (293 [HEK-293] cells, ATCC CRL-157) were maintained in Dulbecco's Modified Eagle's Medium (DMEM) supplemented with 250 U/ml gentamicin and 10% heat-inactivated FCS (Life Technologies, USA). 24 hours prior to transfection, 4 × 10^5^ cells were seeded into a 6-well plate in 3 ml growth medium to reach 70–80% confluency at the time of transfection. 2 μg plasmid DNA was diluted in serum-free DMEM and 4 μl of TurboFect transfection reagent (Thermo Scientific, USA) was added. The mix was incubated for 20 minutes at room temperature and then added to the cells. 24 hours later, the supernatant was examined for released protein. Stable transfected cells were selected using G418 (800 μg/ml) beginning with day 2 after transfection and resistant cells were continuously expanded. To confirm protein expression and release, supernatants were dialyzed against 0.5 M NaCl, 25 mM NaPO_4_, pH 7.0 and protein was isolated using NiNTA agarose beads (Qiagen, Germany). The beads were incubated for 4 hours under constant shaking, washed three times with buffer and gp41 was eluted with 0.5 M NaCl, 25 mM NaPO_4_, 300 mM imidazole, pH 7.0.

### Large-scale production and protein purification

Stably transfected HEK 293 cells were seeded into Corning Roller Bottles (Life Sciences, USA) and supernatants containing recombinant gp41 were collected. Using crossflow filtration (Sartorius, Germany), about 10 litres of supernatant per batch were concentrated 10-fold and diafiltrated to 25 mM NaPO_4,_ pH 7.0. Protein purification was performed using NiNTA agarose beads in a stirred batch procedure, capturing gp41 in the presence of 20 mM imidazole and 0.5 M NaCl overnight at 4°C. The beads were then loaded on a column, washed with buffer containing 50 mM imidazole and gp41 was released by applying a washing buffer supplied with 300 mM imidazole. Protein concentration in these semi-purified preparations was measured by Western Blot analysis using serial dilutions of a bacterially expressed gp41 antigen and 2F5 as primary antibody. Suitable fractions were dialyzed against PBS and incubated for two hours with protein G beads, previously crosslinked to the human monoclonal antibody 5F3 using 13 mg/ml dimethyl pimelimidate (DMP) in 0.2 M triethanolamine and PBS. The beads were washed with PBS and bound gp41 was eluted by applying 0.1 M glycine buffer, pH 2.0. One molar Tris buffer, pH 8.0 was added immediately to neutralize the acidic solution. Eluted fractions were analysed via Western Blot and silver staining. Finally, the buffer was exchanged into PBS and the protein concentration was determined using the BCA method (Thermo Scientific, Germany). Although the established purification protocol resulted in pure material and was highly reproducible, overall yields were rather low (20–25 μg/L) as a result of the low levels of secreted material. For biotinylation of the antigen, the EZ-Link Sulfo-NHS-Biotinylation Kit (Thermo Fisher Scientific, Germany) was used as recommended by the manufacturer.

### Glycosylation analysis

Purified gp41 was dissolved in 10 μl of denaturing buffer for five minutes at 95°C and subsequently digested with PNGaseF or EndoH (NEB, Germany) in the respective enzyme buffers for 1 h at 37°C. Sample buffer was added directly to the mixture and the proteins resolved and detected by SDS-PAGE and Western blotting.

### SDS-PAGE and Western blot analysis

If not stated otherwise, samples were separated by denaturing, reducing SDS-PAGE followed by semi-dry transfer of proteins onto a nitrocellulose support (GE Healthcare, USA). The membranes were blocked with TBS-T buffer (Tris buffered saline with 0.05% Tween 20) containing 5% w/v non-fat dry milk, incubated with anti-gp41 mAb 2F5 (0.3 μg/ml, Polymun Scientific, Austria) in TBS-T buffer for two hours and bound primary antibody was detected with an anti-human IgG-HRP conjugate (1:10.000, Dako, Germany) using the Lumi-Light Western Blotting Substrate (Roche, Germany). Chemiluminescence was measured using a G:Box (VWR, USA) imaging system and Syngene's GeneSnap software.

### Native PAGE

Native PAGE was performed using Novex Native PAGE 4–20% Tris-Glycine gradient gels (Life Technologies, USA) along with SERVA Native protein markers (Serva Electrophoresis GmbH, Germany). Gels were run at 100 V in the Novex Tris-Glycine Native Running Buffer (Life Technologies, USA) and transferred to nitrocellulose, stained with Ponceau S to assign marker bands and then subjected to Western blotting using the 2F5 antibody as described above.

### Capture ELISA

Monoclonal anti-gp41 antibodies were diluted in 100 mM carbonate buffer, pH 9.0 to 5 μg/ml and coated in triplicates on ELISA plates (50 μl/well) overnight at 4°C. Unbound material was washed away with PBS containing 0.05% Tween 20 (PBS-T), plates were blocked for one hour in PBS-T containing 3% BSA and washed once again. Subsequently, 100 ng of purified gp41 diluted in PBS, 0.5% BSA was added to each well and incubated for one hour. After washing for five times, bound gp41 was detected by an HRP-conjugated anti-HIS probe (Life technology, USA) and developed using TMB substrate solution (GE Healthcare, USA). All incubation steps were performed either at 4°C or 37°C as indicated.

### Surface plasmon resonance analysis

Affinity measurements were performed on a Biacore X100 device (GE Healthcare, Germany) at 37°C using the human or mouse antibody capture kit and CM5 sensor chips according to manufacturer’s recommendations (GE Healthcare, Germany). Briefly, antibodies (Table 1) were diluted in running buffer (HBS-EP+, pH 7.4) between 1.5 to 10 μg/ml to reach immobilization levels between 300 to 500 resonance units (RU) after 60 seconds injections at a flow rate of 5 μl/min on flow cell 2. Single cycle bindings kinetics were determined by injecting increasing amounts of recombinant gp41 over both flow cells ranging from 4.5 to 364 nM (1:3 dilution series). Kinetic binding rates *k*_a_, *k*_d_ and the equilibrium binding constants K_D_ were determined by fitting double referenced sensorgrams with the 1:1 Langmuir binding algorithm of the Biacore Evaluation software (2.01) [23].

### Binding of gp41 to human PBMCs

Human PBMCs from healthy donors were isolated from buffy coats using Leucosep tubes (Greiner BioOne, Germany) and Histopaque 1077 gradient medium (Sigma-Aldrich, Germany) according to the manufacturer’s instructions. PBMCs were washed twice with PBS and erythrocytes were lysed by incubation with 0.86% ammonium sulphate for 20 minutes at 37°C. After two consecutive washing steps, cells were incubated in RPMI 1640 containing 10% foetal calf serum (FCS, Biochrom, Germany) with 5% CO_2_ in a humidified incubator. Freshly isolated PBMCs were resuspended in RPMI medium to 1×10^7^ viable cells per millilitre. Cells were tested for viability using the Guava ViaCount reagent as recommended by the manufacturer and cells analysed in the Guava Personal Cell Analysis system (Merck Millipore, Germany). In all cases, viability was ≥90%. 1×10^7^ cells were incubated with indicated amounts of biotinylated gp41 for 2 h at 4°C, non-bound material was washed away with PBS and cells were fixed with BS3 (Thermo Fisher Scientific, Germany) for 30 minutes. To detect bound gp41, an AlexaFluor488-Streptavidine conjugate (Thermo Fisher Scientific, 1:1000) was added to the cells for 20 minutes. The washed samples were finally measured in a FACSCalibur device using the CellQuest software (Becton-Dickinson, USA). Data were analysed using the FlowJo Software (Becton-Dickinson, USA). The presented results are representative data from one of two independent experiments performed in triplicates on different donors.

### Endotoxin assays

Endotoxin content in samples was measured using the EndoLISA System (Hyglos, Germany) according to the manufacturer’s instructions. Briefly, LPS standards ranging from 50–0.05 EU/ml or peptide polymers were mixed with binding buffer in duplicates and incubated on LPS-specific phage coated ELISA plates for 2 hours at 37°C under shaking. After washing, recombinant factor C substrate was added and fluorescence was measured after 0 and 90 minutes. Endotoxin content in the samples was close to the detection limit of the assay (0.05 EU/ml) and below 0.2 EU/μg of protein.

### Cytokine array

To analyse cytokine modulations triggered by gp41, cellular supernatants from 1.5x10^6^ human PBMCs from healthy blood donors which has been incubated for 24 hours with endotoxin-free PBS, 5 EU/ml of purified LPS B:O:055 (Hyglos, Germany) or 1 μg/ml of recombinant gp41 were harvested and pre-cleared by centrifugation. The supernatant was added to nitrocellulose membranes spotted with antibodies to 102 human cytokines and chemokines (Proteome Profiler Human XL Cytokine Array Kit, RD Systems, USA) following the instructions of the manufacturer. Membranes were subsequently analysed using the ImageJ Dot blot Analyzer software to digitalise signal intensities. A pixel density of 50.000 was set as minimum threshold for determination of target cytokines modulated by either LPS or gp41. Data were evaluated using the KOBAS 2.0 software [24] and the KEGG Pathway analysis tool [25]

### IL-10 assay

Human PBMCs were isolated as described above and were resuspended in RPMI medium to 3x10^6^ viable cells per millilitre. Samples to be tested were diluted in endotoxin-free PBS, and added to a 96-well microtiter plate (TRP, Germany) along with LPS standards, RPMI and endotoxin-free PBS controls. Subsequently, 100 μl of purified PBMCs (3x10^5^ cells) were added to each well. Supernatants were collected after 24 hours by centrifugation at 2000 g for 10 min and tested by a commercial IL-10 ELISA (BD Biosciences, San Diego, USA). In order to analyse whether 293 cells producing gp41 may directly induce IL-10 in human PBMCs, 5x10^4^ gp41 producing cells were incubated for 24 hrs with 3x10^5^ PBMCs and the supernatant was tested by ELISA.

### OT-1 CD8 T cells and cTRAMP co-culture experiments

Spleen and lymph nodes were taken from OT-1 T cell receptor (TCR) transgenic mice (Charles River, USA), which produce CD8^+^ T cells with a specific TCR against the ovalbumin peptide 257–264 (SIINFEKL) [26]. Single cell suspensions were generated and naïve CD8^+^ T cells were isolated using the MACS cell separation technology (Miltenyi Biotec, Germany). Wild type cTRAMP prostate cancer cells (ATTC CRL-2730) were kindly provided by A. Sada Japp (BCRT Berlin, Germany) and maintained in DMEM 4.5 g/L glucose supplemented with 5% FCS, 4 mM L-glutamine, 1.5 g/L sodium bicarbonate, 0.005 mg/ml bovine insulin and 10 nM dehydroisoandrosterone, 90%, and 5% of Nu-Serum IV (Corning, USA). Cells were transfected with the pIRESneo vector expressing aminoglycoside 3‘-phosphotransferase APH (3‘) II as enzyme conferring the neomycin resistance or the pIRESneo-gp41 construct and grown under selective pressure with G418 (0.5 μg/ml) for two weeks. 0.5x10^6^ naïve CD8^+^ T cells from OT-1 mice were incubated with 1x10^6^ mock-transfected or gp41 expressing cTRAMP cells pulsed with SIINFEKL. 18 hrs post incubation, Brefeldin A (LC Laboratories) was added and 6 hours later cells were analysed by flow cytometry. To exclude dead cells, samples were incubated with Live/Dead Red (Thermo Fisher Scientific, Germany) prior fixation and permeabilisation (BD Biosciences, Germany) followed by subsequent staining for CD8-Alexa700, CD25-PerCP and IFNγ-PECy7 (all Biolegend, Germany). The presented results are representative data from two independent experiments with duplicate measurements.

## Results

### Recombinant gp41 is expressed in human 293 cells and released into the supernatant

**Fig 1:**
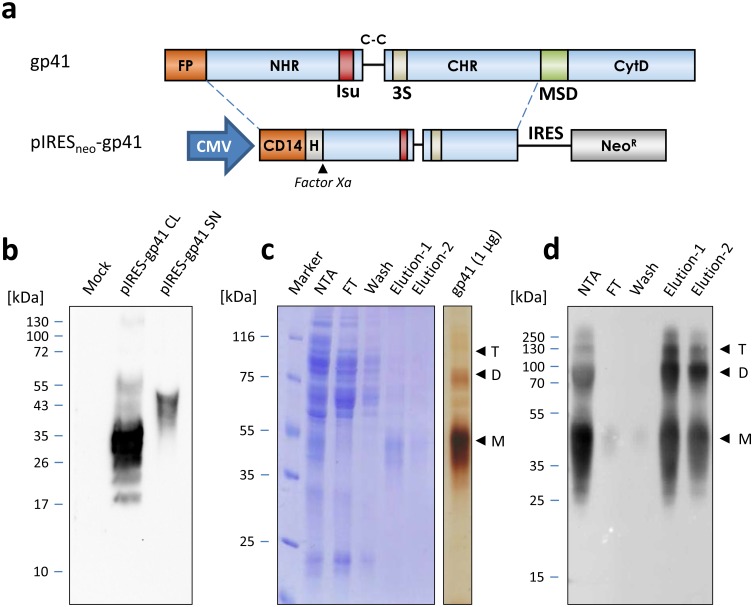
Production and purification of recombinant gp41 expressed in 293 cells. a) Schematical presentation of HIV gp41 and the fragment cloned into the pIRESneo vector to obtain pIRESneo-gp41. The vector is driven by the constitutive CMV promoter (CMV) and triggers expression of gp41 with a CD14 export signal, an N-terminal 8xHis-tag (H) and a factor Xa site in parallel to the neomycin resistance gene (NeoR), which is linked to the gp41 coding sequence by an internal ribosome entry site (IRES). FP, Fusion peptide, NHR, CHR, N- and C-terminal helical region, Isu, immunosuppressive domain, C-C, Cysteine loop, 3S, 3S motif, MSD, membrane spanning domain, CytD, cytoplasmic domain. b) Expression of gp41 in stably selected 293 cells. Lysates of mock transfected cells (mock) or cell lysates (CL) from cells transfected with pIRESneo-gp41 or supernatants from pIRESneo-gp41 cells incubated with NiNTA beads (SN) were separated by SDS-PAGE and immunoblotted, followed by probing with the gp41-specific monoclonal antibody 2F5. Gp41 was specifically detected in cell lysates (CL) as well as cell-free supernatants. c) Tandem-purification of gp41. Coomassie blue stained gel of fractions from cell free supernatants subjected to incubation with NiNTA beads, washing and elution (NTA) followed by a second affinity purification step using the monoclonal antibody 5F3. FT, flow through, wash, PBS wash, elution 1 and 2, fractions after elution using glycine buffer. On the right, 1 μg of purified gp41 was subjected to SDS-PAGE and silver staining to demonstrate purity of the protein. The positions of gp41 monomers (M), dimers (D) and trimers (T) are indicated by an arrow and the molecular weight marker is shown. d) Western blot analysis of the different purification fractions shown in c) after probing of membranes with the antibody 2F5.

To express gp41 in human 293 cells, the sequence of the HIV-1 pNL4-3 ectodomain was amplified by PCR and inserted into a CMV-driven expression vector designated pIRES_neo_-gp41 (Fig 1a).

Through this cloning strategy, a CD14 signal peptide for secretion into the supernatant, an N-terminal His-tag for affinity purification and a factor Xa protease cleavage site was introduced to allow tag removal from the purified protein if necessary. The highly hydrophobic fusion peptide of gp41, the membrane-spanning domain and the cytoplasmic tail, containing two recycling motives for re-internalisation of gp41 from the cell surface, was omitted by this cloning strategy to avoid non-specific aggregation, cytotoxicity issues and to achieve higher levels of secretion. Moreover, the protein was expressed from a bicistronic mRNA encoding gp41 and the neomycin resistance gene by using an IRES element to allow for selection of double positive cells only. After successful transfection of the construct pIRESneo-gp41, expression of the recombinant protein was observed in the transfected 293 cells (Fig 1b). A main protein with a molecular weight of 35 kDa was present in the cytoplasm, whereas the protein secreted into the supernatant of the cells had a molecular weight of 54 kDa, indicating a higher form of glycosylation. After stable cell lines expressing gp41 were established, G418 was titrated on the cells to balance protein production and cell growth, which resulted in doubling of protein yields at 1.5 μg/ml of G418 (S1 Fig). Cells were expanded and gp41 was produced in large-scale using roller bottles. Subsequently, purification of gp41 was performed under non-denaturing conditions using a tandem affinity purification approach. First, bulk contaminants were removed by NiNTA purification followed by a second affinity purification step using the monoclonal antibody 5F3 (Fig 1c). Whereas the material eluted from the NiNTA column still contained various contaminating proteins, gp41 obtained from the 5F3 affinity column was highly pure as confirmed by SDS- PAGE and Coomassie blue staining as well as sensitive silver staining (Fig 1c). Notably, even in the presence of an ionic detergent, purified gp41 formed dimers and trimers during SDS- PAGE (Fig 1c). This was consistent with previous findings showing that gp41 found in infected cells or freshly prepared virus lysates has a high tendency to multimerise [27]. Western blot analyses using the mAb 2F5 confirmed these results, clearly showing that monomers, dimers and trimers were present (Fig 1d).

### Purified recombinant gp41 is glycosylated and trimeric

**Fig 2:**
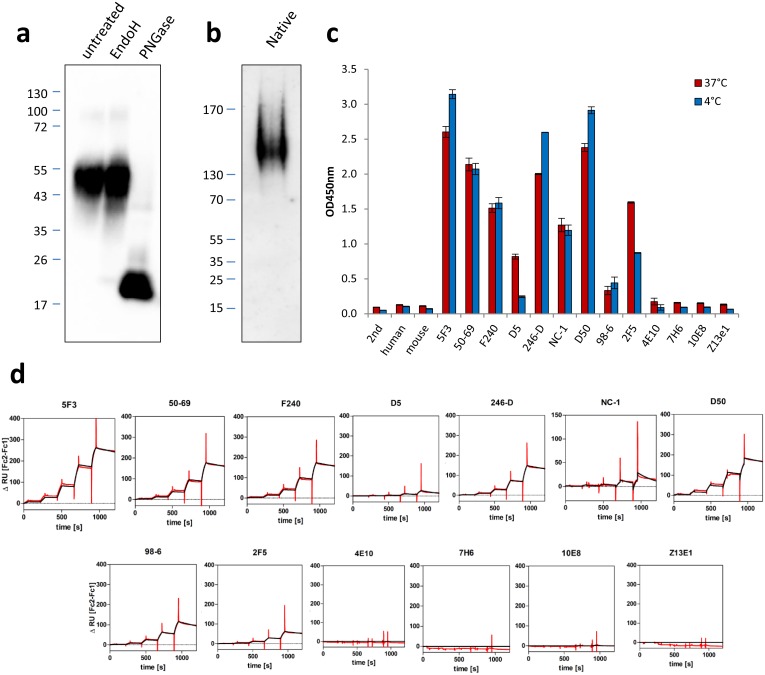
Biochemical and immunological characterization of purified gp41. a) Glycosylation analysis of purified gp41. The protein was denatured and left untreated or incubated with Endoglycosidase H (EndoH) or with PNGase F (PNGase) as indicated. The exclusive sensitivity to PNGase F digestion suggests that the protein is properly post-translationally modified with N-linked sugar residues. b) Analysis of the purified protein using native PAGE, The migration of gp41 at ~160 kDa suggests a trimeric state of the protein. c) Binding of different monoclonal antibodies to recombinant gp41. Binding was measured in a capture ELISA at 4 or 37°C incubation temperature with indicated antibodies coated to the plate. 2nd, secondary anti HIS-HRP probe only, human, anti-gp120 control antibody, mouse, anti gp120 control antibody. For details on the monoclonal antibodies, please see Table 1. Different levels of binding and a temperature sensitive recognition pattern was observed for selected antibodies. d) Results of surface plasmon resonance studies using recombinant gp41 and various monoclonal antibodies. Single cycle kinetics were determined by capturing the indicated anti-gp41 antibodies to the chip via covalently linked anti-mouse or anti human IgG, followed by five injections of gp41 at increasing concentrations. Data shown (ΔRU) represent double referenced sensograms with relative signal intensities obtained by subtraction of flow cell 2 (gp41 antibody) and flow cell 1 (no antibody). Measurement curves (black) and fitted curves (red) are indicated.

To further characterise the produced recombinant gp41, protein glycosylation was studied in a next step. The expressed gp41 contained four predicted N-linked glycosylation sites at amino acid positions 616, 625, 637, and 674, whereas O-linked glycosylation sites were absent. In line with this, only treatment of the purified protein with Glycosidase F (PNGase F) which cleaves between the innermost GlcNAc and asparagine residues of high mannose, hybrid, and complex oligosaccharides from N-linked glycoproteins but not endoglycosidase H, which removes high mannose N-glycans, resulted in a mobility shift from ~54 kDa down to approximately 20 kDa during SDS-PAGE (Fig 2a).

This result was in line with the predicted molecular weight of the native protein chain (19.8 kDa) and indicated that N-linked glycosylation is the major post-translational modification of the protein. However, the loss of approximately 34 kDa by the glycosidase treatment was larger than expected for four glycosylation sites (~16 kDa) and might be explained by glycan structures with higher complexity [28]. In comparison to the previous SDS-PAGE results, electrophoresis of gp41 using a native gel demonstrated the presence of only a single band migrating below the 170 kDa size marker, supporting a trimerised state (~160 kDa) of the protein under non-denaturing conditions (Fig 2b).

### Conformation specific antibodies indicate a six-helix bundle conformation of gp41

To elucidate the structure of the purified antigen, a set of monoclonal antibodies specific for gp41 were used. In first experiments, binding of gp41 was analysed in a non-denaturing capture-ELISA setup, in which the antigen was added to the ELISA plate previously coated with one of the selected monoclonal antibodies (Table 1). Strong to intermediate reactivity was observed for the antibodies 5F3, 50–69, F240, 246-D, NC-1, D50 and 2F5 (Fig 2c). However, antibodies D5 and 98–6 bound only weakly to the antigen. Furthermore, the MPER-directed antibodies 4E10, 7H6, 10E8 and Z13e1 were reacting at baseline levels comparable to the gp120-directed or secondary antibody controls (Fig 2c). Interestingly, there were differences in antigen capture when the assay was performed at high and low temperatures and this effect was most pronounced in the case of antibodies D5, D50 and 2F5. The prefusion/intermediate state specific antibodies D5 and 2F5 showed reduced capture of gp41 at 4°C, whereas binding of the 6HB reactive antibody D50 was increased (Fig 2c). This reciprocal recognition pattern was indicative for a strong 6HB formation at low temperatures, with a more relaxed 6HB/intermediate structure at 37°C, which might result from increased Brownian motion at this temperature. These data were confirmed and extended by surface plasmon resonance experiments using the same panel of antibodies (Fig 2d, Table 1). Consistent with the ELISA results, most of the MPER-reactive bnAbs including 4E10, 7H6, and 10E8 were not binding to the antigen, indicating that the MPER epitope is not readily accessible in the gp41 antigen. In contrast, the mAb 5F3 bound tightly to the antigen (*K*_D_ = 2.8 nM), with fastest on-rate (*k*_a_ 1.1×10^5^) and lowest off-rate (*k*_d_ 3.0×10^−4^) in the panel (Table 1). Since this antibody recognises a linear epitope in the MPER, but preferentially binds to gp41 oligomers, these data supported the PAGE results above, suggesting that the gp41 antigen is a trimer. Considering the conformation-specific antibodies in the panel, highest affinities were measured with D50 and the cysteine loop-dependent antibody 50–69, followed by antibodies 98–6, 2F5, NC-1 and D5 (Table 1). As in the ELISA experiments, about 3.5-fold higher affinity of the 6HB-specific mAb D50 (*K*_D_ = 5.1 nM) compared to prefusion-reactive 2F5 (*K*_D_ = 18 nM) was observed. Taken together, these binding data provided sufficient evidence to conclude that the gp41 antigen produced here is in a disulphide-bridged state and its predominant conformation is the 6HB.

### Recombinant gp41 binds mainly to monocytes

**Fig 3:**
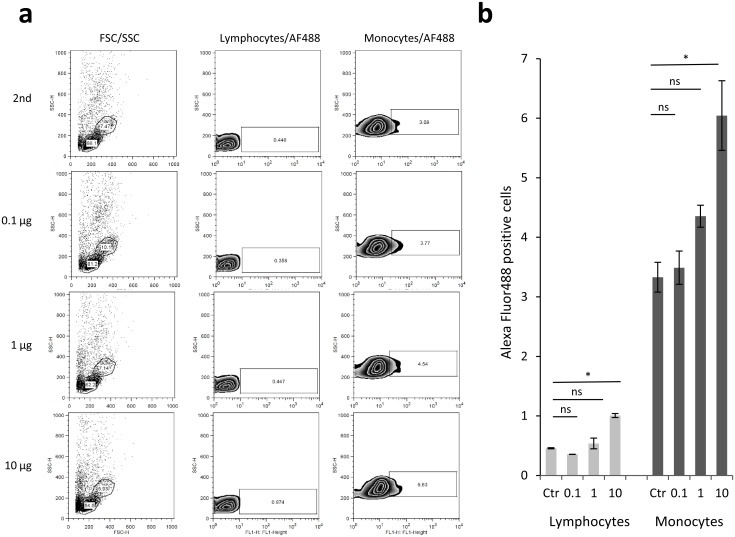
Binding of purified and biotinylated gp41 to human PBMCs. a) Freshly isolated PBMCs were mixed with no or increasing amounts of gp41 (0.1, 1 and 10 μg) followed by washing and crosslinking after 2 hours at 4°C. Subsequently, bound gp41 was detected by addition of an AlexaFluor488-Streptavidine conjugate (2nd) using FACS analysis. Monocytes and lymphocytes were stratified based on the forward/sideward scatter profile (FSC/SSC gate) and fluorescence measured in the FL1 channel. A dose-dependent binding to lymphocytes and monocytes was observed in this representative experiment. b) Quantification of binding to human lympho- and monocytes with the same experimental setup as in a) in three replicate measurements.

In previous studies, gp41 has been suggested to be involved in immunopathogenesis by inhibiting mitogen-triggered lymphoproliferation, modulating gene expression and cytokine release on human immune cells [12]. In order to evaluate whether these effects might be triggered by a direct interaction, a biotinylated form of purified gp41 was co-incubated with freshly isolated PBMCs at low temperatures to prevent an unspecific uptake. Thereafter, cells were washed and fixed and bound gp41 was detected with a fluorescent streptavidin conjugate. In the following FACS analysis, a dose-dependent binding to monocytes (3–6%) and to a lesser extend also to lymphocytes (0.5–1%) was observed (Fig 3).

### Recombinant gp41 triggers cytokine release in human PBMCs

**Fig 4:**
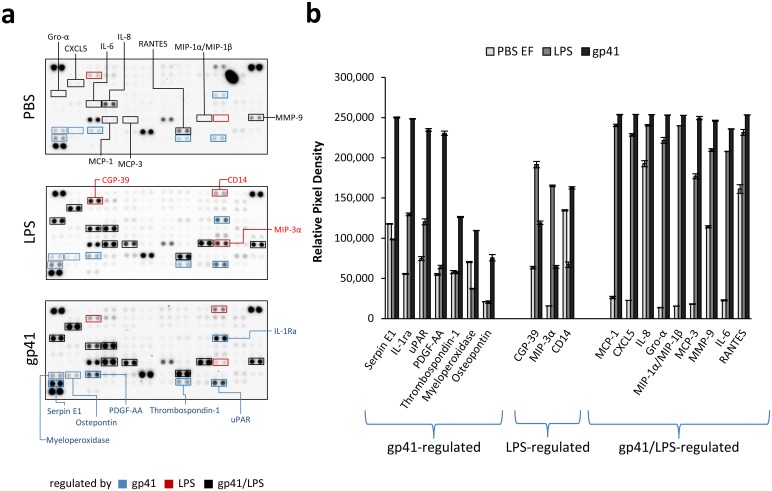
Modulation of cytokine release by recombinant gp41. a) A cytokine array was performed with supernatants of PBMCs previously incubated with PBS, 5 EU/ml of LPS or 1 μg of purified gp41 for 24 hours. Cytokines specifically altered by incubation with LPS, gp41 or both LPS and gp41 are framed in red, blue and black, respectively. b) Analysis of modulated cytokine patterns after densitometric scanning of the membranes in a) was performed to quantify changes induced by gp41, LPS or gp41 as well as LPS.

Next, we were interested to investigate if even such comparatively weak interaction with human immune cells was sufficient to induce changes in cytokine production. Therefore, human PBMCs were incubated with purified gp41 having a low endotoxin content (see below) and after 24 hours, supernatants were analysed for 102 different cytokines, chemokines and growth factors using a cytokine array. Additional samples comprised PBS and LPS, which were included as negative and positive controls, respectively. Whereas several of the cytokines were marginally affected or not altered at all (S2 Fig), a distinct subset of cytokines regulated by gp41, or LPS could be identified (Fig 4).

In case of gp41, this included up-regulation of SerpineE1 (also called Nexin,Plasminogen Activator Inhibitor Type 1), interleukin 1 receptor antagonist (IL-1Ra), urokinase receptor (uPAR or CD87), platelet-derived growth factor (PDGF-AA, composed of two A chains), thrombospondin-1 (THBS-1), myeloperoxidase (MPO) as well as osteopontin (OPN), a small integrin binding ligand N-linked glycoprotein. In a subsequent KEGG analysis (Table 2), these cytokines mapped to different pathways including that of focal adhesion and PI3K-Akt signalling (THBS-1, PDGF, OPN, p = 0.0049 and p = 0.0192, respectively), the complement and coagulation cascade (SerpineE1, uPAR, p = 0.006) as well as the phagosome (THBS-1, MPO, p = 0.0266).

**Table 2:** KEGG Pathway analysis of cytokines induced in human PBMCs after incubation with gp41.

Pathway Name	Pathway ID	Targets	P-Value
Focal adhesion	hsa04510	THBS-1, PDGFAA, OPN	0.0049
p53 signaling pathway	hsa04115	SerpineE1, THBS-1	0.0058
Complement and coagulation cascades	hsa04610	SerpineE1, uPAR	0.0060
ECM-receptor interaction	hsa04512	OPN, THBS-1	0.0093
PI3K-Akt signaling pathway	hsa04151	THBS-1, PDGFAA, OPN	0.0192
Phagosome	hsa04145	THBS-1, MPO	0.0266
Transcriptional misregulation in cancer	hsa05202	PDGFAA, MPO	0.0354
Proteoglycans in cancer	hsa05205	THBS-1, uPAR	0.0443
Rap1 signaling pathway	hsa04015	PDGFAA, THBS-1	0.0475

THBS-1, thrombospondin-1; PDGF-AA, platelet-derived growth factor, composed of two A chains; uPAR, urokinase receptor or CD87; MPO, myeloperoxidase

In the LPS control group, upregulation of CGP-39 (chitinase-3-like protein 1), macrophage inflammatory protein 3α (MIP-3α, CCL20) and down-regulation of soluble CD14, which is part of the LPS receptor, was observed. Interestingly, some cytokines including monocyte chemotactic protein 1 (MCP-1, also CC-chemokine ligand 2, CCL2), C-X-C motif chemokine 5 (CXCL5), IL-8, growth-regulated alpha protein (Gro-α, or CXCL1), macrophage inflammatory proteins MIP-1α/MIP-1β, monocyte-specific chemokine 3 (MCP-3, or CCL7), matrix metallopeptidase 9 (MMP-9), IL-6 and RANTES (regulated on activation, normal T cell expressed and secreted, also called chemokine (C-C motif) ligand 5, CCL5) were similarly regulated by LPS and recombinant gp41. Together, these results demonstrated that the presence of gp41 actively influenced the cytokine production of human PBMCs, with certain cytokines being specifically regulated by gp41.

### Influence of the recombinant gp41 on IL-10 release

**Fig 5:**
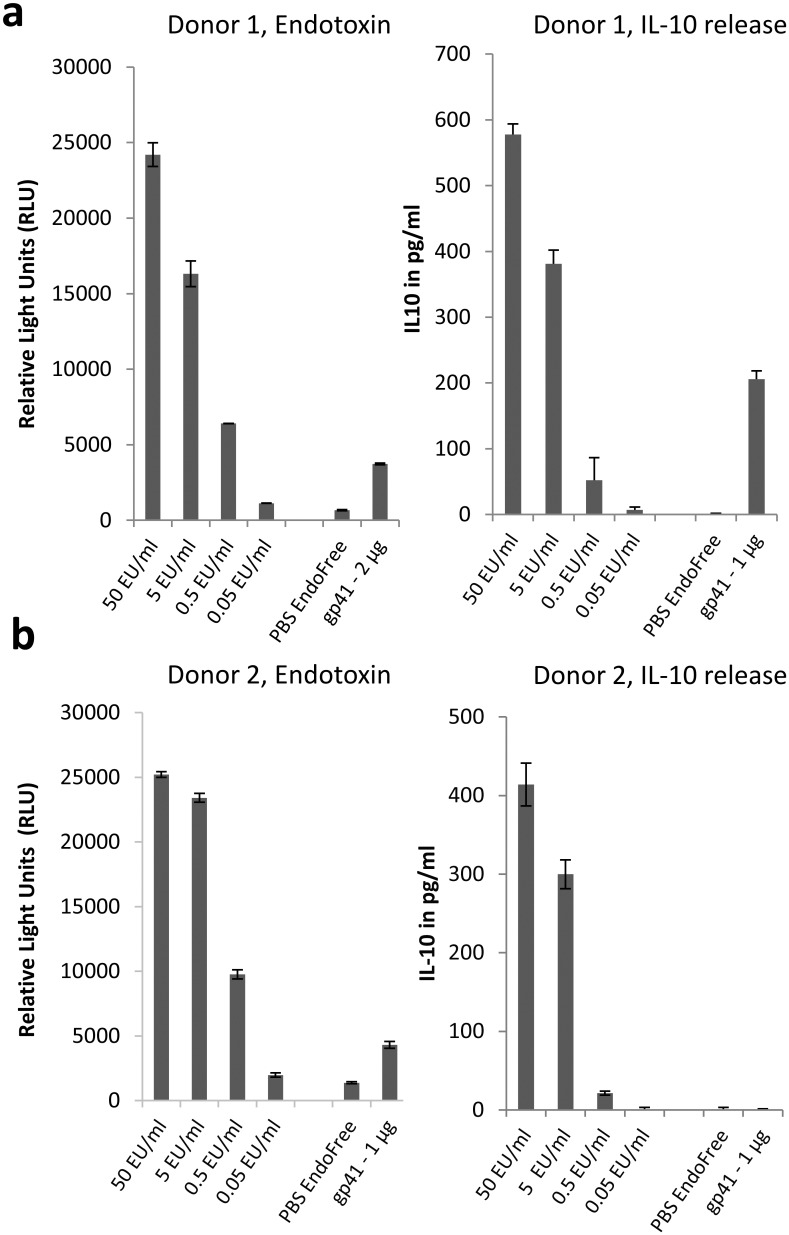
Ability of recombinant endotoxin-free gp41 to induce IL-10. a) Determination of the endotoxin content of the gp41 preparation (left) and its ability to induce IL-10 in isolated PBMCs (right) from donor 1. b) Determination of the endotoxin content of the gp41 preparation (left) and its ability to induce IL-10 in isolated PBMCs (right) from donor 2. Although identical amounts of antigen with comparable endotoxin content were applied in both assays, IL-10 induction was not consistently detected.

In the cytokine array, differences in the expression of IL-10 could not be observed. This finding was somewhat unexpected, since at least LPS is well established as a strong inducer of IL-10 secretion [29] and gp41 had also been reported to induce IL-10 [17, 18]. We hypothesised, that the lack of IL-10 detection might be a result of lower sensitivity of the antibody pair used in the cytokine array compared to other commercially available assay systems and thus re-evaluated IL-10 expression in separate experiments. As before, the individual gp41 preparations were tested for their LPS content and found to contain very low endotoxin levels close to the detection limit of the assay (Fig 5a and 5b, left panel). When added to PBMCs of two different donors, robust and comparable levels of IL-10 were induced with the included LPS standard, however, gp41 was able to trigger only low levels of IL-10 in one of the two donors (Fig 5a and 5b, right panel).

### Recombinant gp41 inhibits CD8^+^ T cells and is interspecies-reactive

**Fig 6:**
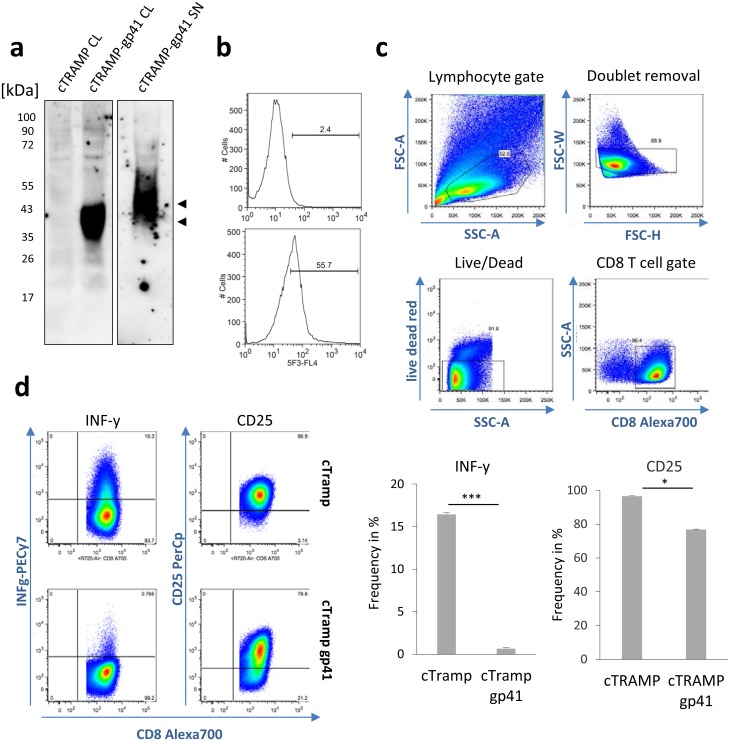
Recombinant gp41 expressed on murine tumour cells inhibits the effector functions of antigen-primed CD8^+^ T cells. a) Analysis of gp41 expression and secretion in stably selected cTRAMP cells. Lysates of empty vector (cTRAMP CL) or gp41 plasmid transfected cTRAMP cells (cTRAMP-gp41 CL) or supernatant of cTRAMP-gp41 cells incubated with NiNTA beads were separated by SDS-PAGE and immunoblotted with the gp41-specific monoclonal antibody 2F5. Similar to transfected 293 cells, gp41 was detected in cell lysates and secreted into the supernatant. b) FACS analysis of pIRESneo vector transfected cTRAMP cells (top) and pIRESneo-gp41 vector transfected cTRAMP-gp41 cells (bottom) using the gp41-specific antibody 5F3 confirms substantial levels of surface expression. c, d) Inhibition of IFNγ and CD25 expression by CD8^+^ T cells from OT-1 mice co-cultured with pIRESneo vector transfected or gp41 expressing cTRAMP cells pulsed with SIINFEKL peptide. The gating strategy to retrieve viable CD8^+^ T cells in the mixture is shown in c). Results are representative data from two independent experiments with duplicate measurements.

To investigate for the first time the immunosuppressive activity of gp41 on the T cell immunity, murine cTRAMP prostate cancer cells were stably transfected with the pIRESneo vector expressing only APH (3‘) II as enzyme conferring the neomycin resistance or with the gp41-expressing pIRESneo vector. As in the case of the 293 cells used for production, gp41 expressing cTRAMP cells expressed the protein in the cell lysate and released larger proteins characterised by higher glycosylation into the supernatant (Fig 6a).

The expression of gp41 on the cell surface was also demonstrated by FACS analysis using the monoclonal antibody 5F3 (Fig 6b). After pulsing these cells with the ovalbumin-derived MHC-1 peptide SIINFEKL, they were used for co-cultivation experiments with naïve CD8^+^ T cells from OT-1 mice carrying the corresponding SIINFEKL T cell receptor. By analysing markers for activation and effector function in CD8^+^ T cells 18 hours later, the gp41 expressing cells, but not the APH (3‘) II expressing cells, induced a strong inhibition of IFNγ production (Fig 6d, p = 0.0001, Two-paired Student´s T-Test, 95% CI). Similarly, CD25 expression was impaired (Fig 6d, p = 0.01, Two-paired Student´s T-Test, 95% CI). These data demonstrated that the expression of gp41 inhibits expression of IFNγ as an important mediator of antiviral immunity and of the IL-2 receptor in CD8^+^ T cells. The experiment was performed with an expression vector to make sure expression on the cell surface und direct interaction with the putative receptor on the CD8^+^ T cells. This experiment also confirmed previous results showing that the transmembrane envelope proteins or peptide corresponding to their immunosuppressive domain act on the immune cells from other species (for review see [12]).

## Discussion

In the present study, a recombinant gp41 of HIV-1 was produced in human cells and its antigenic and immunosuppressive properties were investigated. After confirming protein integrity and proper glycosylation, comprehensive efforts were undertaken to properly characterise the antigen. This was considered particularly important, since studies with synthetic peptides corresponding to the immunosuppressive domain showed that the conformation obtained by the preparation of homopolymers or conjugation to a carrier protein is important for the biological activity [17, 30, 31]. Based on its behaviour during SDS and native PAGE as well as the monoclonal antibody binding pattern (Figs 1 and 2), we conclude that the gp41 antigen produced here is in a trimeric state and has a postfusional 6HB conformation. Interestingly, binding of distinct mAbs to recombinant gp41 was found to be dependent on the assay temperature. This temperature sensitivity is consistent with results obtained by other groups, demonstrating the existence of a temperature arrested state for gp41 [32] that can increase the potency of NHR-specific neutralising antibodies by prolonging the time frame for antibody access [33, 34]. Concerning neutralization, except for 2F5 there was low affinity of all MPER-targeting bnAb in the panel. Whereas in case of 4E10 this might be explained by a lack of lipids [35], it is somewhat unexpected in case of the bnAbs 7H6 and 10E8, since all critical residues for binding are actually present in the antigen [36]. Together, this implies that the gp41 antigen produced here would not perform very well in immunization studies aiming to induce MPER-specific bnAbs, due to its inability to bind and stimulate B cells with a matching B cell receptor. However, it is difficult to predict the potential conformation of gp41 in an oily adjuvant when using it for immunization.

The produced recombinant gp41 was glycosylated and it would be interesting to study the influence of glycosylation on lymphocyte binding and immunosuppressive properties. However, since synthetic peptides corresponding to the Isu domain were able to inhibit lymphocyte proliferation and to modulate cytokine release and gene expression [17, 30, 37] and were binding to PBMCs [21, 22], we suggest that glycosylation is not required for binding and immunosuppressive activities.

The purified gp41 was able to interact with human PBMCs, particularly with the monocyte fraction (Fig 3). The finding that the gp41 binds to monocytes and to a lesser extent to lymphocytes confirms recent data showing that homopolymers of peptides corresponding to the Isu domain of gp41 did bind predominantly to human classical and intermediate monocytes and B cells [22]. Similar to the previous investigations, an uptake via endocytosis or by the LPS (TLR4/CD14/MD2) receptor could be excluded through the experimental set-up: performing the incubation at 4°C and showing that the preparation did not contain LPS. Compared to this former study, however, the level of binding observed with gp41 was only moderate (6% compared to 45% on monocytes and 1% compared to 10% on lymphocytes). It is currently unclear, whether this is caused by a less effective binding of the larger gp41 molecule to the cells, whether the introduction of the biotin-tag to lysine residues of the antigen might has sterically blocked potential interacting domains, or whether the reduced temperature used during incubation to prevent unspecific uptake might restrict the structural flexibility of the molecule potentially needed for interaction. Differences in the conformation had been shown here at different temperatures (Fig 2), indicating gp41 may change its conformation. Next to this, it will be interesting to investigate gp41 binding on a larger cohort of PBMC donors to evaluate whether donor-dependent differences exist. Based on previous results obtained with the gp41 immunosuppressive peptide [22], this might be anticipated.

Despite the low level of binding, gp41 induced cytokine expression in human PBMCs, with a distinct subset being exclusively regulated by gp41 but not LPS (Fig 4). The advantage of the gp41 produced here in human 293 cells in contrast to many other gp41 produced in bacteria is that it is free of endotoxin. Some of the up-regulated cytokines and genes had been reported previously after incubation of homopolymers of peptides corresponding to the Isu domain with human PBMCs, but this study is a significant extension of the previous study [17]. Among the proteins reported for the first time, IL-1Ra represents a well-known immunosuppressive cytokine able to inhibit the pro-inflammatory responses stimulated by binding of IL-1β to the IL-1 receptor [38, 39]. Interestingly, gp41 also upregulated THBS-1, PDGFA and OPN expression, which stimulates actin remodelling through integrin-mediated signalling via Src, focal adhesion kinase (FAK) and PI3K. Although further data is needed to prove this, it is tempting to hypothesise that gp41 might be involved in the spontaneous filopodia formation at the virological synapse seen in HIV-1 infected cells [40]. Supporting this, another positive regulator of FAK signaling, CD63, has been recently described to physically interact with gp41 [41]. The association of gp41 induced cytokines with the complement pathway seems likely, since gp41 has been shown to induce NKp44L expression by binding the complement receptor gC1qR on CD4^+^ cells via its 3S domain [42]. It has been shown that apoptosis mediated by Env glycoprotein in bystander cells in fact correlates with gp41-induced hemifusion and this involves reactive oxygen species production [43] and gp41-induced NO formation was thought to contribute to the severe cognitive dysfunction associated with HIV-1 infection [44]. The enhanced expression of myeloperoxidase observed here corroborates this data and substantiates the association with the p53 pathway. Although in this study no gp41 mutated in the Isu domain was included, in a previous study abrogation of the cytokine inducing activity of gp41 by single mutations in the Isu domain was clearly demonstrated [18].

The recombinant gp41 obtained here was unable to consistently induce IL-10 release (Fig 5). This contradicts previous studies showing IL-10 induction using recombinant gp41 expressed and purified from *E*. *coli* [45–48] but also results obtained with synthetic peptides corresponding to the Isu domain [17], gp41 produced in eukaryotic cells or using purified HIV-1 [18]. A strong induction of IL-10 was also reported for the transmembrane envelope protein of other retroviruses [49, 50]. In the past, we have co-cultured HIV-1 producing T cell lines (expressing full length HIV-Env), human teratocarcinoma or melanoma cell lines expressing Env of the human endogenous retrovirus HERV-K as well as human cells producing the porcine endogenous retrovirus PERV (also expressing full length PERV-Env) with PBMCs from normal human donors and did not observe IL-10 release (unpublished data). At present, we generated cells expressing small recombinant proteins containing the immunosuppressive domain of HIV-1 and PERV and incubation of these cells with PBMCs from healthy human donors led to IL-10 release (unpublished). Together these data support the view that the conformation and accessibility of Isu is critical for its biological function and that the amount presented on cells in the context of full length Env might be not sufficient to induce IL-10 production. The latter observation could result from shielding through the surface protein. Obviously, the 6HB conformation of the gp41 used here is not the most effective to induce this effect. Further production of the ultrapure gp41 may allow analysing why the gp41 induces IL-10 release only in rare cases. Conditions modifying the conformation of the trimeric gp41 may also help to answer this question.

Intriguing and new is the finding, that the expression of gp41 inhibits directly T cell immunity. Gp41 expressed on peptide pulsed target cells inhibited the expression of IFNγ and CD25 by antigen-specific cytotoxic CD8^+^ T cells. CD25 is the alpha chain of the IL-2 receptor expressed on activated T cells and regulatory T cells. Mutations in this alpha chain are the cause of a severe immunodeficiency called severe combined immunodeficiency (SCID). Although the co-incubation of the target cells with the specific immune T cells is an *in vitro* experiment, it reminds *in vivo* studies by Heidmann et al. [13–16], showing that tumour cells not growing into a tumour in immunocompetent mice, do so when a retroviral transmembrane envelope proteins is expressed on their cell surface. This was shown for several retroviral transmembrane proteins and deletions or mutations in the immunosuppressive domain abrogated the immunosuppressive effect [16, 18]. This experiment also confirms the often reported interspecies-reactivity of the retroviral transmembrane envelope proteins or peptides corresponding to their immunosuppressive domain (for review see [12]). In summary, our data provide another line of evidence that gp41 might be directly involved in HIV-1 immunopathogenesis through modulation of the cytokine release and active inhibition of immune responses.

## Supporting information

S1 Fig Optimisation of gp41 expression under selective pressure. Stably transfected 293 cells were pre-cultured for 3 days under the indicated G418 concentrations and then seeded at equal cell numbers into cell culture dishes. After another three days, supernatants were recovered and incubated with NiNTA beads to enrich secreted gp41 and corresponding cell lysates were prepared in parallel. 20 μg of cell lysates and gp41 eluted from beads by boiling were separated by SDS-PAGE and after transfer to nitrocellulose incubated with the monoclonal antibody 2F5. Increasing the G418 concentration to 1.5 μg/ml approximately doubled to yield of gp41 in the supernatant and was thus selected for large-scale production. Marker lanes and associated molecular weights are indicated on the left and the right. Arrows indicate the intracellular (IC) or secreted, glycosylated (SG) form of gp41, respectively.
(TIF)

S2 Fig Overview of cytokines, chemokines and growth factors modulated in human PBMCs after incubation with PBS, LPS or recombinant gp41. The cut-off at a relative pixel density of 50.000 is visualised by a slashed line.
(TIF)
