# Supplementary figures and images for: Antigenic and immunosuppressive properties of a trimeric recombinant transmembrane envelope protein gp41 of HIV-1
Source: PLoS One. 2017 Mar 10;12(3):e0173454. doi: 10.1371/journal.pone.0173454 (PMC5345815; doi:10.1371/journal.pone.0173454)

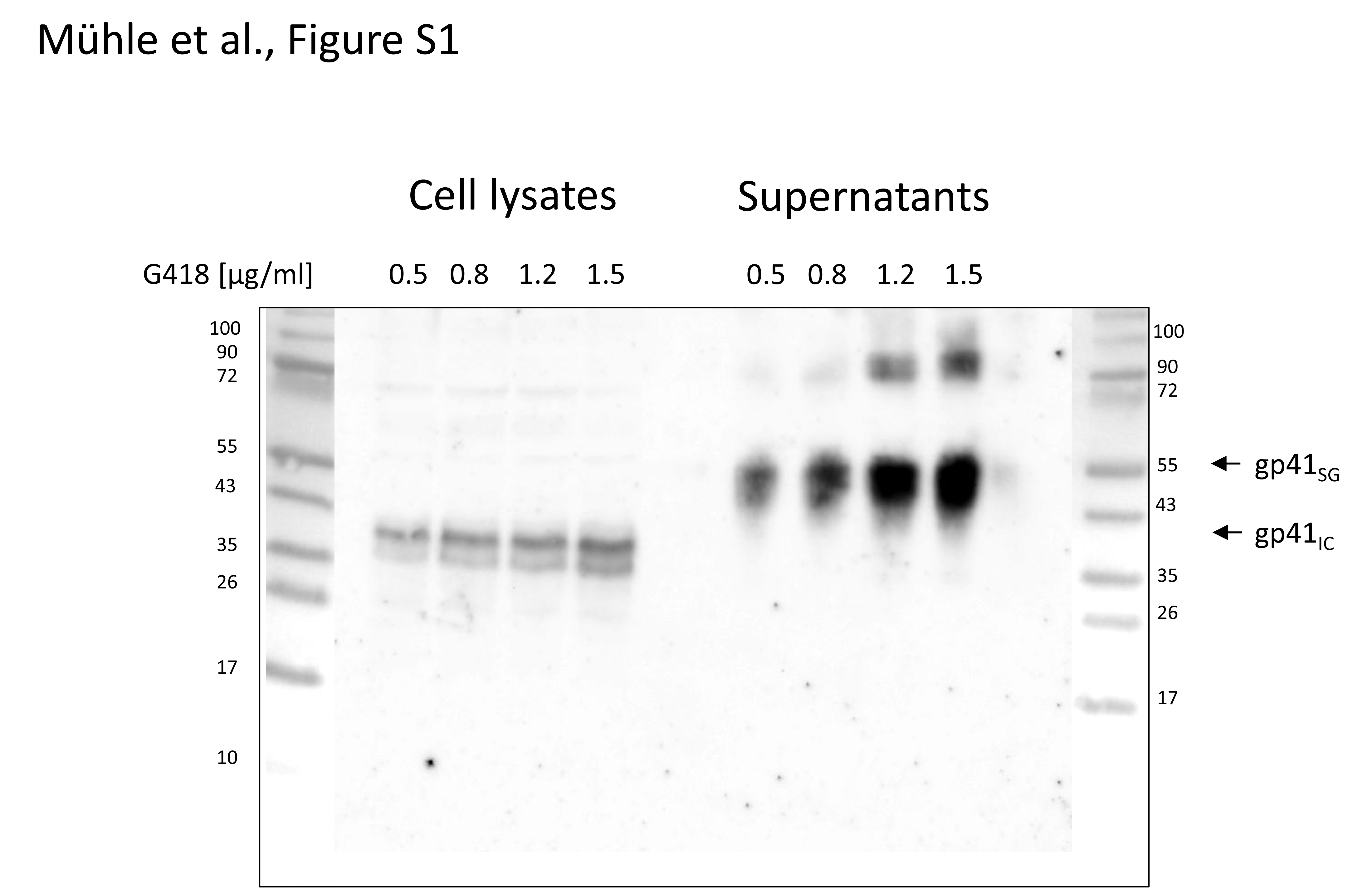

Supplement: S1 Fig — Stably transfected 293 cells were pre-cultured for 3 days under the indicated G418 concentrations and then seeded at equal cell numbers into cell culture dishes. After another three days, supernatants were recovered and incubated with NiNTA beads to enrich secreted gp41 and corresponding cell lysates were prepared in parallel. 20 μg of cell lysates and gp41 eluted from beads by boiling were separated by SDS-PAGE and after transfer to nitrocellulose incubated with the monoclonal antibody 2F5. Increasing the G418 concentration to 1.5 μg/ml approximately doubled to yield of gp41 in the supernatant and was thus selected for large-scale production. Marker lanes and associated molecular weights are indicated on the left and the right. Arrows indicate the intracellular (IC) or secreted, glycosylated (SG) form of gp41, respectively. (TIF) [file pone.0173454.s001.tif]
